# Comparison of the effectiveness of nurse-led preoperative counseling and postoperative follow-up care vs standard care for patients with gastric cancer

**DOI:** 10.1515/med-2024-1098

**Published:** 2025-01-17

**Authors:** Wenwen Wang, Yan Zhu, Yu Sun, Yandong Li

**Affiliations:** Department of Cardiovascular Medicine, The Affiliated Hospital of Beihua University, Jilin, Jilin, China; School of Nursing, Beihua University, East Campus of Beihua University, Jilin City, Jilin, China; Department of General Surgery, The Affiliated Hospital of Beihua University, Jilin City, Jilin, China; Department of General Surgery, The Affiliated Hospital of Beihua University, No. 12 Jiefang Middle Road, Chuanying District, Jilin City, Jilin, China

**Keywords:** aftercare, gastric cancer, healthcare professional-led follow-up care, mortality, nurse, preoperative counseling, prognosis, radical gastrectomy, surgeon

## Abstract

**Background:**

Radical gastrectomy is generally prefered for gastric cancer but has postoperative complications. The objectives of the study are to evaluate the effectiveness of three different models of preoperative counseling and postoperative follow-up care in patients who underwent radical gastrectomy for gastric cancer.

**Methods:**

In retrospective medical record analyses, patients received nurse-led preoperative counseling and postoperative follow-up care (NC cohort, *n* = 105) or surgeon-led preoperative counseling and surgeon-led follow-up (SC cohort, *n* = 140), or did not receive counseling and aftercare (RC cohort, *n* = 160).

**Results:**

Patients had a postoperative intensive care unit (PICU) stay of 24 (26–22) h/patient and postoperative pain of 6 (6–5)/ patient. The frequencies of nausea, vomiting, medical intensive care unit admission(s), dizziness, length of PICU stay, and intensity of postoperative pain were lower among patients in the NC cohort, followed by those in the SC and RC cohorts. A higher number of patients in the RC cohort died when compared to the NC (25 [16%) vs 2 [2%], *p* = 0.0458) and the SC (25 [16%] vs 11 [8%], *p* = 0.0001) cohorts in follow-up.

**Conclusion:**

Patients who undergo radical gastrectomy for gastric cancer require healthcare professional-led preoperative counseling and postoperative aftercare. Nurse-led preoperative counseling and postoperative aftercare, could improve outcome measures in patients who underwent radical gastrectomy for gastric cancer.

## Graphical abstract

**Figure:**
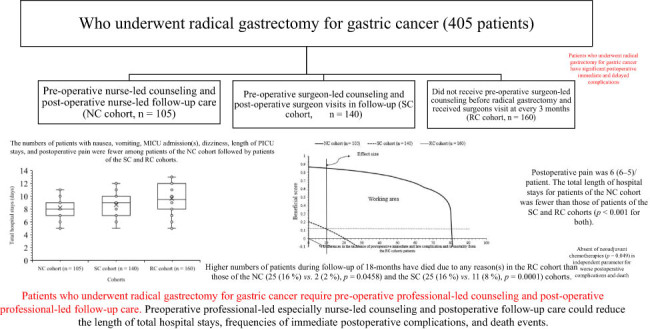

## 1 Introduction

More than one million new cases of gastric cancer were reported worldwide in 2020. In addition, half of all cancer patients in China have gastric cancer [1]. Because of the diet conditions of the Chinese population, the number of patients with gastric cancer is higher in the Chinese population than in the Australian, American, or European populations [2]. Radical gastrectomy is generally preferred in patients with gastric cancer [3]. However, postoperative complications and mortality are frequent due to issues associated with the radical gastrectomy procedure [4]. Postoperative complications of radical gastrectomy are quite common, and some are rare, but they have a clinical impact in practice [5,6]. Postoperative complications for radical gastrectomy cause poor prognosis, and patients may have a longer hospital stay [7]. By managing the frequency of postoperative complications for radical gastrectomy, it is possible to improve prognosis and reduce the length of hospital stay and mortality events in patients with gastric cancer after a gastrectomy procedure [7].

Preoperative therapies and/or aftercare also have a positive impact on postoperative prognosis, length of hospital stay, and mortality in patients with gastric cancer after radical gastrectomy [8,9]. Moreover, preoperative instructions to patients before surgery decrease their anxieties and cancellation of surgeries [10]. Nurse-led care is more cost-effective and has a positive effect on postoperative prognosis than consultant-led care [11]. Moreover, a retrospective study [12] reported that nurse-supervised exercises at the institute are associated with a decreased frequency of complications that cause rehospitalization and death in patients with acute pancreatitis.

The objectives of this retrospective study are to evaluate the frequencies of postoperative complications, length of hospital stay, and number of death events in the northeast region of China among patients with gastric cancer who underwent radical gastrectomy and received preoperative nurse-led counseling and postoperative nurse-led follow-up care against standard care (preoperative surgeon-led counseling and postoperative surgeon-led aftercare) against those who did not receive preoperative health professional-led counseling before radical gastrectomy and received only surgeons visit every 3 months.

## 2 Materials and methods

### 2.1 Research design

The research design consists of a retrospective study using patients’ medical records.

### 2.2 Inclusion criteria

Patients (aged ≥18 years or more) who underwent elective radical gastrectomy (laparoscopy or laparotomy) were included in the study.

### 2.3 Exclusion criteria

Patients with cognitive impairments and those with records of three or more vital parameters missing from the patients’ records of the institute(s) were excluded from the analyses.

### 2.4 Sample size calculations

The statistical analyses for sample size calculation were carried out using the XLSTAT trial version, Excel Statistical Software, Addinsoft, New York, NY, USA, and based on the assumption that the incidence of postoperative immediate and late complications and/or mortality of patients who underwent radical gastrectomy would be decreased 10% with preoperative healthcare professional-led counseling and postoperative professional-led care than those who did not receive preoperative healthcare professional-led counseling before radical gastrectomy and received surgeons visit in follow-up at every 3 months, 5% margin of error(s), 95% confidence interval (CI), and 10% of standard deviation (SD), the sample size (minimum patients required in a cohort) would be 105. Kruskal–Wallis’ test was used for the sample size calculation.

### 2.5 Cohorts

A total of 105 patients received preoperative nurse-led counseling, postoperative nurse-led follow-up care between chemoradiotherapy and follow-up after radical gastrectomy, and surgeon(s) visits every 3 months during follow-up (NC cohort). A total of 140 patients received preoperative surgeon-led counseling before radical gastrectomy, and the surgeon(s) visited every 3 months between chemoradiotherapy and during follow-up after radical gastrectomy (SC cohort). A total of 160 patients did not receive preoperative surgeon-led counseling before radical gastrectomy and underwent surgeon(s) visits every 3 months between chemoradiotherapy and during follow-up after radical gastrectomy (RC cohort). The selection of healthcare professional-led care was based on the availability of professionals in the institutes and choice of patients. Availability was based on empty appointment spots in the future (patient could wait for 1 or 2 weeks).

### 2.6 Details about the operation method

All the enrolled cases are advanced upper one-third or esophageal gastric junction cancers and all cases in the cohort underwent total gastrectomy. Mostly patients underwent subtotal or the extent of lymph node dissection, and the gastrointestinal reconstruction method was used.

### 2.7 Non-treatment interventions

#### 2.7.1 Preoperative nurse-led counseling and postoperative nurse-led follow-up care

Preoperative nurse-led counseling included a discussion of the risks and benefits of radical gastrectomy, including cost factors for patients and caregivers. The nurse used visual aids to describe the situation after surgery. Postoperative nurse-led care included visits by nurses every 15 days. The surgeon visits the institute every 3 months. Postoperative nurse-led follow-up care included aftercare regarding postoperative adverse effects and mortality, including education and instructions for food habits. The duration of the visit of the surgeon(s) and nurse(s) was a maximum of 30 min. However, they were available via phone calls.

#### 2.7.2 Standard care (preoperative surgeon-led counseling and postoperative surgeon-led care)

Preoperative surgeon-led counseling included a discussion of the risks and benefits of radical gastrectomy with patients and caregivers. Postoperative surgeon-led care included visits by the surgeon(s) at the institute every 3 months. Postoperative surgeon-led care included care regarding postoperative adverse effects and mortality, including education and instructions regarding food habits. The maximum length of visits by the surgeon(s) was 30 min. However, they were available via phone calls.

Patients in the RC cohort received literature on postoperative care. The selection of patients in the NC, SC, and RC cohorts was based on the availability of professionals in the institute. Patients in the NC and SC cohorts received healthcare professional-led aftercare for a minimum of 6 months.

### 2.8 Outcome measures

#### 2.8.1 Demographic and clinical parameters

Demographic, clinical, treatment, and surgery-related parameters were extracted from the patients’ medical records and analyzed.

#### 2.8.2 Postoperative complications

After surgery, immediate and delayed postoperative complications in the postoperative intensive care unit (PICU), ward, and follow-up of 6 months were collected from the hospital records of patients and analyzed. Clavien-Dindo classification method was preferred for evaluation of immediate complications after radical gastrectomy [13].

#### 2.8.3 PICU stays

From completion of operation to transfer to ward.

#### 2.8.4 Total hospital stays

After completion of the operation to discharge of patient.

#### 2.8.5 Death

The death of patient due to any reason(s) was considered death (mortality) during 18 months of follow-up.

#### 2.8.6 Clinical benefits for healthcare professional-led counseling and professional-led aftercare

The clinical benefits of healthcare professional-led counseling and aftercare of patients who underwent radical gastrectomy for gastric cancer were evaluated as a function of the beneficial scores. Beneficial scores for healthcare professional-led counseling and healthcare professional-led aftercare were calculated from the risk of undercare, as expressed in equation (1). The risk of undercare was defined by a calculation that involved the incidence of immediate and late postoperative complications and/or mortality of patients who underwent radical gastrectomy for gastric cancer (equation (2)). The confidence for radical gastrectomy for gastric cancer was considered a numerical value from 0 to 1. The beneficial score of the healthcare professional-led counseling and healthcare professional-led aftercare is the area above the curve of the counseling and care adopted, and the working area is the area under the curve of the adopted counseling and care methods. For all adopted counseling and aftercare, 10% or more of differences in the incidence of postoperative immediate and late complications and/or mortality of patients (with respect to patients of the RC cohort) were used as the reference standard [14].

(1) \[{\mathrm{Beneficial\; score}}=\frac{{\mathrm{Number\; of\; patients\; with}}10 \% {\mathrm{or\; more\; of\; the}}{\mathrm{differences\; in\; the}}{\mathrm{incidence\; of\; postoperative\; immediate\; and\; late\; complication\; and}}/{\mathrm{or\; mortality}}}{{\mathrm{Total\; number\; of\; patients}}}-\left(\frac{{\mathrm{Number\; of\; patients\; with\; less\; than}}10 \% {\mathrm{of}}{\mathrm{the}}{\mathrm{differences\; in\; the}}{\mathrm{incidence\; of\; postoperative\; immediate\; and\; late\; complication\; and}}/{\mathrm{or\; mortality}}}{{\mathrm{Total\; number\; of\; patients}}}\times {\mathrm{Risk\; of\; under\; care}}\right),]\]

(2) \[{\mathrm{Risk\; of\; under\; care}}=\frac{{\mathrm{The\; differences\; in\; the\; incidence\; of\; postoperative\; immediate\; and\; late\; complication\; and}}/{\mathrm{or\; mortality}}}{100-{\mathrm{the}}{\mathrm{differences\; in\; the}}{\mathrm{incidence\; of\; postoperative\; immediate\; and\; late\; complication\; and}}/{\mathrm{or\; mortality}}}.]\]

### 2.9 Statistical analysis

InStat 3.01 GraphPad Software (San Diego, CA, USA) was used for the statistical analysis. Calculator Soup^®^ (https://www.calculatorsoup.com/calculators/statistics/quartile-calculator.php) was used to calculate the quartile values. Normally distributed continuous variables, non-normally distributed continuous variables, and categorical variables are presented as mean values ± SD, medians (Q3–Q1), and frequencies (percentages), respectively. The Kolmogorov and Smirnov methods were used to check the normality of continuous variables or data. The unpaired *t*-test, paired *t*-test, or one-way analysis of variance (ANOVA) was preferred for normally distributed continuous variables. For non-normally distributed continuous variables, the non-parametric Wilcoxon test, Mann–Whitney U test, or Kruskal–Wallis’ test was used. Dunn’s test was used for *post hoc* analysis of non-normally distributed continuous variables. Tukey’s test was used for *post hoc* analysis of normally distributed continuous variables. Bartlett’s test was used to check assumption of unpaired *t*-test and ANOVA. Levene’s test was used for homogeneity of variances for Wilcoxon signed-rank test. The chi-square test or Fisher’s exact test was preferred for statistical analyses of categorical variables. Multivariate analyses (logistic regression analyses) were performed to evaluate the association between demographic and clinical parameters and worse postoperative complications in patients [8]. All results were considered significant if the *p*-value was less than 0.05 at 95% CI.


**Ethical approval:** The protocols for retrospective medical record analyses were designed by the authors themselves and approved by the Beihua University review board (Approval number YDZJ202201ZYTS156 dated January 15, 2020) and the Chinese Anti-Cancer Association. The study followed the law of China and the v2008 Declarations of Helsinki.
**Informed consent:** Consent to participate was waived by the Beihua University review board because of the retrospective medical record analyses.

## 3 Results

### 3.1 Study population

**Figure 1:**
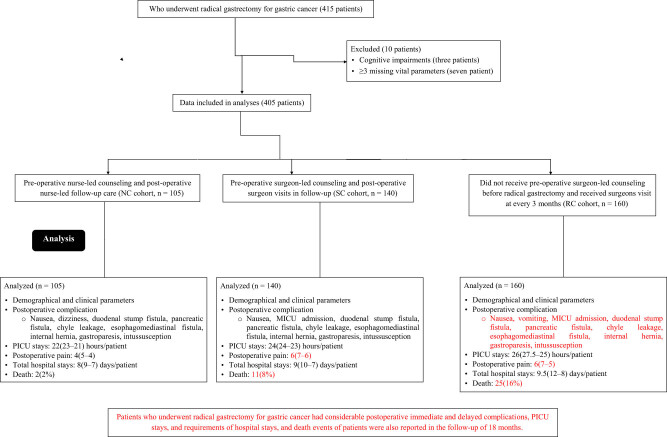
The chart of retrospective medical record analyses. PICU: Postoperative intensive care unit, MICU: Medical intensive care unit. Red color indicates worse parameters. Black color indicates not good but not worse parameters.

From January 15, 2020 to July 31, 2022, a total of 415 patients with gastric cancer underwent radical gastrectomy at the Affiliated Hospital of Beihua University, Jilin City, Jilin Province, China, and the referring hospitals. Among them (415 patients), three patients had cognitive impairments, and three or more vital parameters were missing from the hospital patient records of seven patients. Therefore, the data from these ten patients were excluded from the analyses. Data on postoperative complications, PICU stay, total hospital stay, and death in 405 patients who underwent radical gastrectomy for gastric cancer were included in the study. A chart of the retrospective medical record analysis is presented in Figure 1.

### 3.2 Demographic and clinical parameters

A total of 73% of patients were male. Patients in the range of 48–63 years were included in the analyses. Most of the patients were Han Chinese. One-third of radical gastrostomies were laparoscopies. Only 12% of patients received neoadjuvant chemotherapy. The surgery time was between 150 and 208 min/patient. The body mass index (BMI) of the patients was between 24 and 25.8 kg/m^2^. Sex, age, ethnicity, nature of gastrectomy, preference for chemotherapy, BMI, and surgery time for patients were comparable among cohorts (*p* > 0.05 for all). The demographic and clinical parameters are reported in Table 1. All patients included in the study were subjected to adjuvant therapy.

**Table 1:** Demographic, clinical, and surgical parameters of the enrolled patients

Parameters	Total	Cohorts					
		NC	SC	RC	Comparison between cohorts		
Professional-led Care	–	Preoperative nurse-led counseling and postoperative nurse-led follow-up care	Preoperative surgeon-led counseling and postoperative surgeon visits in follow-up	Follow-up surgeons’ visits/3 months only			
Number of patients	405	105	140	160	*p*-value	df	Test value
Gender							
Male	295 (73)	70 (67)	105 (75)	120 (75)	0.2554 (*χ* ^2^-test for independence)	2	2.73
Female	110 (27)	35 (33)	35 (25)	40 (25)			
Age (years)	54 (59–51)	54 (58–51)	55 (60–51)	54 (59–51)	0.1059 (Kruskal–Wallis’ test)	N/A	4.49
Ethnicity							
Han Chinese	371 (92)	96 (91)	129 (92)	146 (91)	0.9955 (*χ* ^2^-test for independence)	4	0.1951
Mongolian	31 (7)	8 (8)	10 (7)	13 (8)			
Tibetan	3 (1)	1 (1)	1 (1)	1 (1)			
Nature of gastrectomy							
Laparoscopy	150 (37)	40 (38)	50 (36)	60 (38)	0.9184 (*χ* ^2^-test for independence)	2	0.1702
Laparotomy	355 (63)	65 (62)	90 (64)	100 (62)			
Neoadjuvant chemotherapies							
Yes	49 (12)	11 (10)	17 (12)	21 (13)	0.8111 (*χ* ^2^-test for independence)	2	0.4186
No	356 (88)	94 (90)	123 (88)	139 (87)			
Surgery time (min)	186 (195–170)	178 (190.5–170)	188 (196–169.5)	187 (195.5–168)	0.1454 (Kruskal–Wallis’ test)	N/A	3.586
BMI (kg/m^2^)	25.7 (26–24.9)	25.5 (25.9–24.8)	25.8 (26–25)	25.8 (26–25)	0.1082 (Kruskal–Wallis’ test)	N/A	4.447
Education							
Primitive	69 (17)	24 (23)	22 (16)	23 (14)	0.2688 (*χ* ^2^-test for independence)	6	7.601
Up to higher secondary	180 (44)	36 (34)	65 (46)	79 (49)			
Higher secondary but below graduate	116 (29)	32 (30)	41 (29)	43 (27)			
Graduate or more	40 (10)	13 (13)	12 (9)	15 (10)			

Non-normal distributed continuous variables and constant variables are depicted as medians (Q3–Q1) and frequencies (percentages).

All results were considered significant if the *p*-value was less than 0.05 at 95% CI.

df: Degree of freedom, N/A: not applicable, BMI: Body mass index, CI: confidence interval.

Test value (*χ* ^2^-value for *χ* ^2^-test; Kruskal–Wallis’ statistics for Kruskal–Wallis’ test).

### 3.3 Outcome measures

#### 3.3.1 Postoperative complications

The number of patients with nausea, vomiting, medical intensive care unit (MICU) admission(s), dizziness, PICU stays, and postoperative pain was fewer among patients of the NC cohort followed by patients of the SC and those of the RC cohorts. The PICU stay was 24 (26–22) h/patient. Postoperative pain was 6 (range, 6–5) per patient. Rare serious early postoperative complications were reported among patients. Details of early postoperative complications are reported in Table 2. Late postoperative complications were reported in patients in all cohorts. There were one, and only one patient with each type of late complication in the NC cohort and seven patients in the RC cohort except eight with pancreatic fistula and internal hernia. There were statistically insignificant differences in each of the late postoperative complications among the patients in all cohorts. However, the number of patients with late postoperative complications was lower among patients in the NC cohort, followed by patients in the SC and RC cohorts. None of the patients reported gastroparesis. Details of the late postoperative complications are reported in Table 3.

**Table 2:** Immediate complications after radical gastrectomy

	Cohorts											
Events	RC	NC				SC						
Care	Follow-up surgeons’ visits/3 months only	Preoperative nurse-led counseling and postoperative nurse-led follow-up care	Comparison between the RC and the NC cohorts			Preoperative surgeon-led counseling and postoperative surgeon visits in follow-up	Comparison between the RC and the SC cohorts			Comparison between the SC and the NC cohorts		
Number of patients	160	105	*p*-value	Test value	95% CI	140	*p*-value	Test value	95% CI	*p*-value	Test value	95% CI
Number of patients with nausea	25 (16)	2(2)	0.0001 (Fisher exact test)	1.632	1.399–1.904	11 (8)	0.0495 (Fisher exact test)	1.358	1.061–1.738	0.0458 (Fisher exact test)	0.3465	0.09603–1.250
Number of patients with vomiting	18 (11)	0 (0)	<0.0001 (Fisher exact test)	1.739	1.563–1.936	6 (4)	0.0324 (Fisher exact test)	1.458	1.126–1.887	0.0389 (Fisher exact test)	0	−Infinite to +Infinite
Number of patients required MICU admission(s)	20 (13)	0 (0)	<0.0001 (Fisher exact test)	1.75	1.570–1.95	7 (5)	0.0264 (Fisher exact test)	1.444	1.123–1.857	0.021 (Fisher exact test)	0	−Infinite to +Infinite
PICU stay (hours)	26 (27.5–25)	22 (23–21)	<0.001 (Kruskal–Wallis’ test/Dunn’s test)	272.83	N/A	24 (24–23)	<0.001 (Kruskal–Wallis’ test/Dunn’s test)	272.83	N/A	<0.001 (Kruskal–Wallis’ test/Dunn’s test)	272.83	N/A
Number of patients with dizziness	9(6)	1(1)	0.0943 (Fisher exact test)	1.52	1.207–1.914	4(3)	0.2705 (Fisher exact test)	1.316	0.9010–1.922	0.3955 (Fisher exact test)	0.4615	0.07945–2.681
Postoperative pain	6 (7–5)	4 (5–4)	<0.001 (Kruskal–Wallis’ test/Dunn’s test)	131.82	N/A	6 (7–6)	<0.001 (Kruskal–Wallis’ test/Dunn’s test)	131.82	N/A	<0.001 (Kruskal–Wallis’ test/Dunn’s test)	131.82	N/A
Number of patients with fever	5 (3)	2 (2)	0.7068 (Fisher exact test)	1.189	0.7364–1.920	2 (2)	0.455 (Fisher exact test)	1.35	0.8347–2.184	<0.0001 (Fisher exact test)	68.667	17.334–272.01
Number of patients with dumping syndrome	1 (1)	1 (1)	0.9999 (Fisher exact test)	0.827	0.2061–3.319	1(1)	0.9999 (Fisher exact test)	0.9371	0.2333–3.764	0.9999 (Fisher exact test)	1.168	0.2899–4.709
Number of patients with aspiration pneumonia	1 (1)	1 (1)	0.9999 (Fisher exact test)	0.827	0.2061–3.319	1(1)	0.9999 (Fisher exact test)	0.9371	0.2333–3.764	0.9999 (Fisher exact test)	1.168	0.2899–4.709
Number of patients with septic shock	1 (1)	1 (1)	0.9999 (Fisher exact test)	0.827	0.2061–3.319	1 (1)	0.9999 (Fisher exact test)	0.9371	0.2333–3.764	0.9999 (Fisher exact test)	1.168	0.2899–4.709

Non-normal variables and ordinal data are depicted as medians (Q3–Q1). Categorial variables are depicted as frequencies (percentages).

All results were considered significant if the *p*-value was less than 0.05 at 95% CI (using the approximation of Katz for categorical variables).

df: Degree of freedom, N/A: not applicable, CI: confidence interval, MICU: Medical intensive care unit, PICU: Postoperative surgical care unit, In: Infinity.

Test value (*χ* ^2^-value for *χ* ^2^-test; relative risk for Fisher exact test).

A visual analog scale was used to evaluate postoperative pain (evaluated 1 day after surgeries; 0: absent pain, 10: worst possible pain).

**Table 3:** Late complications after radical gastrectomy

	Cohorts											
Events	RC	NC				SC						
Care	Follow-up surgeons’ visits/3 months only	Preoperative nurse-led counseling and postoperative nurse-led follow-up care	Comparison between the RC and the NC cohorts			Preoperative surgeon-led counseling and postoperative surgeon visits in follow-up	Comparison between the RC and the SC cohorts			Comparison between the SC and the NC cohorts		
Number of patients	160	105	*p*-value	RR	95% CI	140	*p*-value	RR	95% CI	*p*-value	RR	95% CI
Number of patients with duodenal stump fistula	7 (4)	1 (1)	0.1517	1.47	1.110 –1.946	2 (1)	0.1819	1.479	1.026 –2.133	0.9999	0.7756	0.1555–3.870
Number of patients with pancreatic fistula	8 (5)	1 (1)	0.092	1.479	1.163 –1.927	3 (2)	0.2295	1.383	0.9474–2.018	0.6373	0.5793	0.1054–3.184
Numbers of patients with chyle leakage	7 (4)	1 (1)	0.1517	1.47	1.110 – 1.946	4 (3)	0.5515	1.202	0.7589–1.904	0.3955	0.4615	0.07945–2.681
Number of patients with esophagomediastinal fistula	7 (4)	1 (1)	0.1517	1.47	1.110–1.946	2 (1)	0.1819	1.479	1.026–2.133	0.9999	0.7756	0.1555–3.870
Number of patients with internal hernia	8 (5)	1 (1)	0.092	1.479	1.163–1.927	3 (2)	0.2295	1.383	0.9474–2.018	0.6373	0.5793	0.1054–3.184
Number of patients with small bowel paresis	7 (4)	1 (1)	0.1517	1.47	1.110–1.946	4 (3)	0.5515	1.202	0.7589–1.904	0.3955	0.4615	0.07945–2.681
Number of patients with intussusception	7 (4)	1 (1)	0.1517	1.47	1.110–1.946	2 (1)	0.1819	1.479	1.026–2.133	0.9999	0.7756	0.1555–3.870

Variables are depicted as frequencies (percentages).

Fisher exact test was used for statistical analysis.

All results were considered significant if the *p*-value was less than 0.05 at 95% CI.

df: Degree of freedom, N/A: not applicable, CI: confidence interval, RR: Relative risk.

#### 3.3.2 Total hospital stays

**Figure 2:**
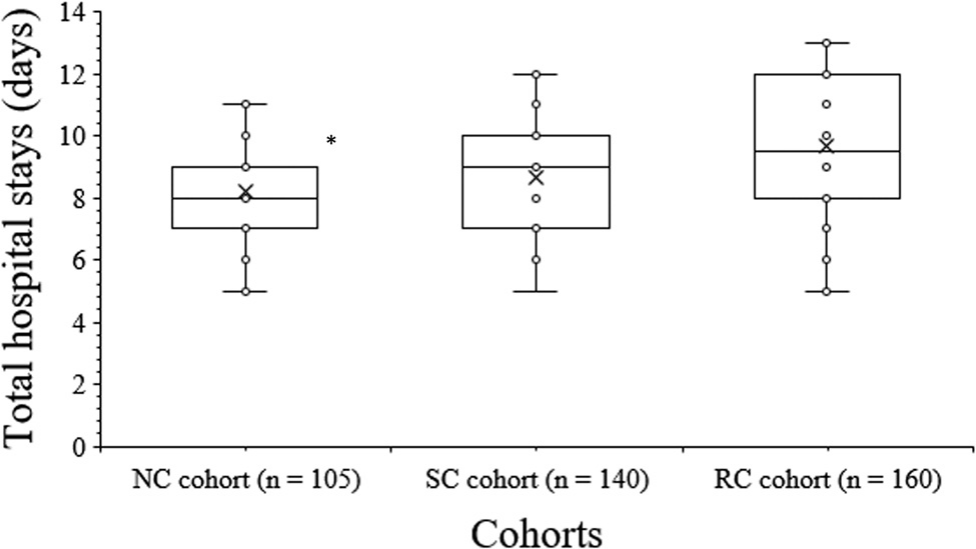
Total hospital stays of patients. Total hospital stays: After completion of operation to discharge of patients. Upper whisker: maximum value, upper box line: third quartile value, *x*: median value, lower box line: first quartile value, lower whisker: minimum value. ^*^: Fewer than those of patients of the SC and RC cohorts.

A total of 8 (9–7) days/patient, 9 (10–7) days/patient, and 9.5 (12–8) days/patient were the total length of hospital stays for patients of the NC, SC, and RC cohort, respectively. The total length of hospital stays for patients in the NC cohort were lesser than those of patients in the SC and RC cohorts (*p* < 0.001 for both, Kruskal–Wallis’ test/Dunn’s test). The total hospital stay for patients in the SC and RC cohorts was statistically similar (*p* > 0.05, Kruskal–Wallis’ test/Dunn’s test). The details of the total length of hospital stay of the patients are shown in Figure 2.

#### 3.3.3 Death

A total of 2 (2%), 11 (8%), and 25 (16%) patients in the NC, SC, and RC cohorts, respectively, died due to some reason(s) during the 18 months of follow-up. During the 18-month follow-up period, a higher number of patients in the RC cohort died for several reasons than those in the NC (25 [16%] vs 2 [2%], *p* = 0.0458, Fisher exact test) and the SC (25 [16%] vs 11 [8%], *p* = 0.0001, Fisher exact test) cohorts. During the 18-month follow-up period, a higher number of patients in the SC cohort died for several reasons than those in the NC cohort (*p* = 0.0495, Fisher exact test).

#### 3.3.4 Independent parameters for worse postoperative complications

The absence of neoadjuvant chemotherapy (*p* = 0.049, logistic regression analysis) was associated with worse postoperative complications and death. The details of the association of demographic and clinical parameters with worse postoperative complications in patients are presented in Table 4.

**Table 4:** Multivariate analyses association of the demographic and clinical parameters with worse postoperative complications of patients

Parameter	Odds ratio	% CI	*p*-value
Gender (female vs male)	0.8142	0.7214–0.8421	0.5621
Age (≥60 years vs <60 years)	0.3254	0.2245–0.3824	0.0821
Nature of gastrectomy (laparoscopy vs laparotomy)	0.7745	0.6541–0.8142	0.3224
Neoadjuvant chemotherapies (^*^No vs yes)	1.4521	1.2141–2.1451	0.049
Surgery time (<200 min vs ≥200 min)	0.9341	0.8142–0.9522	0.0651
BMI (>25 vs ≤25)	0.6654	0.5142–0.8422	0.062

BMI: Body mass index, CI: Confidence interval.

^*^Significant parameter, *p* value less than 0.05, and odds ratio more than 1 considered significant.

#### 3.3.5 Clinical benefits for healthcare professional-led counseling and professional-led aftercare

**Figure 3:**
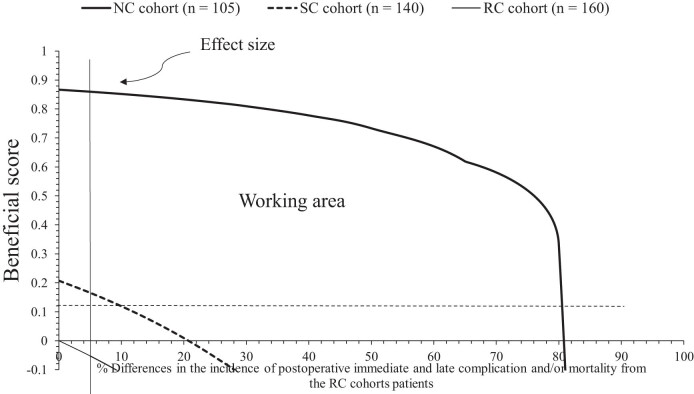
The clinical benefits for healthcare professional-led counseling and healthcare professional-led aftercare.

The beneficial score for patients of the NC cohort had 10–80.8% differences in the incidence of postoperative immediate and late complications and/or mortality, whereas SC cohort had 10–20% differences in the incidence of postoperative immediate and late complications and/or mortality, and RC cohort had no beneficial score for counseling and aftercare for gastric cancer. Above 80.8, 20, and 0% differences in the incidence of postoperative immediate and late complications and/or mortality of patients of the NC, SC, and RC cohorts had the risk of undercare, respectively. The details of the clinical benefits of healthcare professional-led counseling and healthcare professional-led aftercare are presented in Table 5. The graphical presentation of the clinical benefits of healthcare professional-led counseling and healthcare professional-led aftercare are presented in Figure 3.

**Table 5:** Beneficial score analyses

% differences in the incidence of postoperative immediate and late complication and/or mortality from the RC cohort patients	Cohorts		
	NC cohort	SC cohort	RC cohort
Professional-led care	Preoperative nurse-led counseling and postoperative nurse-led follow-up care	Preoperative surgeon-led counseling and postoperative surgeon visits in follow-up	Follow-up surgeons’ visits/3 months only
Numbers of patients	105	140	160
0	0.87	0.21	0
5	0.86	0.17	−0.05
10	0.85	0.12	−0.11
15	0.84	0.07	−0.18
20	0.83	0.01	−0.25
25	0.82	−0.06	−0.33
30	0.81	−0.13	−0.43
40	0.78	−0.32	−0.67
50	0.73	−0.59	−1
65	0.62	−1.27	−1.86
80	0.33	−2.96	−4
99	−12.33	−78.29	−99
Beneficial score (% differences in the incidence of postoperative immediate and late complication and/or mortality from the RC cohort patients)	11–80.8%	10–20%	None
Risk of undercare (% differences in the incidence of postoperative immediate and late complication and/or mortality from the RC cohort patients)	>80.8%	>20%	>0%

Effect size: 10% differences in the incidence of postoperative immediate and late complication and/or mortality compared from the RC cohort patients.

## 4 Discussion

Patients who underwent radical gastrectomy for gastric cancer had considerable postoperative immediate and delayed complications, PICU stays, and requirements of hospital stays, and death events of patients were also reported in the follow-up of 18 months. The results of the current retrospective medical record analyses were consistent with those of a retrospective review of the Chinese population [7]. Patients who undergo radical gastrectomy for gastric cancer require preoperative healthcare professional-led counseling and postoperative follow-up care.

Immediate postoperative complications were reported to be fewer in the NC and SC cohorts than in the RC cohort. The results of immediate postoperative complications of the current retrospective medical record analyses are consistent with those of a retrospective review of the Chinese population [7], a randomized trial [15], and a systematic review [16]. Preoperative counseling by healthcare professionals decreases immediate postoperative complications in gastric surgery [15]. Preoperative counseling by a healthcare professional is necessary in patients who have undergone radical gastrectomy for gastric cancer.

Late surgical complications were observed across the cohorts. The results of late postoperative complications of the current retrospective medical record analyses are consistent with those of a retrospective review of the Chinese population [7]. Late postoperative complications are mainly due to the surgery itself, patient profile, and other comorbidities [5,6]. Several late postoperative complications are rare. In particular, intussusception has not been observed after upper gastrointestinal resection. Additionally, chyle leakage and internal hernias are rare. In any case, these late postoperative complications are highly related to what has been done during the surgery and are rarely related to counseling or lack thereof [17]. The experience and expertise of surgeons and different surgical techniques could lead to a decrease in late postoperative complications in patients after radical gastrectomy for gastric cancer.

The length of total hospital stay was reported to be higher in the RC cohort. Postoperative complications and their treatments increased the length of total hospital stay of patients undergoing gastric surgery [18]. Professional-led preoperative care, especially nurse-led counseling and postoperative follow-up care, could reduce total hospital stays.

During the follow-up period, fewer patients in the NC and SC cohorts, especially in the NC cohort, died for several reasons. The mortality results of the current retrospective medical record analyses are consistent with a retrospective review of the Chinese population [7], meta-analyses [5,6], and literature reviews [18]. Fewer postoperative complications would lead to fewer deaths in patients after gastric surgeries [18]. In addition, age, surgeries, severity of diseases, and present comorbidities are associated with the death of patients after radical gastrectomy [19]. By reducing postoperative complications and improving the experience and expertise of multidisciplinary approaches and teamwork, including surgeons(s), there is hope to decrease the death of patients who have undergone radical gastrectomy for gastric cancer. The details of the comparative studies on nurse-led aftercare for cancer patients undergoing gastric surgeries/oncology surgeries in different settings are presented in Table 6.

**Table 6:** Comparative studies on nurse-led aftercare for cancer patients undergoing gastric surgeries/oncology surgeries in different settings

Study	Published year	Study population	Sample size (*N*; patients)	Age (years)	Follow-up
Meta-analysis, Guerra et al. [5]	2018	Patients underwent minimally invasive radical gastrectomy	2,000	55–74 years	1–6 months
Retrospective review, Yan et al. [7]	2023	Chinese gastric cancer patients underwent radical gastrectomy	891 (432/461)	62.65 ± 10.62/63.22 ± 11.66	6 months
Randomized trial, Verschuur et al. [11]	2009	Dutch patients after intentionally curative surgery for esophageal or gastric cardia cancer	109 (54/55)	61 ± 7; 61 ± 9	13 months
Retrospective analysis, Huang and Zhang [12]	2024	Chinese patients with acute pancreatitis	381 (123/103/155)	55 (60–50)	6 months
Randomized trial, Samnani et al. [15]	2014	Pakistani patients underwent abdominal surgery	232 (1,131/111)	36.7 ± 8.69/ 37.01 ± 8.43	1 month
Post-study analyses, Stratilatovas et al. [19]	2015	Lithuanian patients underwent gastrectomy for gastric cancer	1,674	>18 years	10 years

The absence of neoadjuvant chemotherapy (*p* = 0.049) was associated with worse postoperative complications and death. Patients' willingness or family decisions regarding chemotherapy: Typically, almost all patients with gastric cancer undergo neoadjuvant chemotherapy. However, in the current study, only 12% patients received neoadjuvant therapy. Notably, many diseases, including cancer and gastric cancer, require a multidisciplinary approach and teamwork. At present, we have to consider confounding factors (as mentioned in the results section) for worse postoperative complications.

Gastroparesis was not reported in any of the patient. The patients have all had total gastrostomies. Therefore, they have no stomach involved and cannot have gastroparesis.

This study has several limitations, such as non-randomized, non-intervention(s), retrospective analyses, and lack of controlled trials. This is unfortunate because the basic hypothesis is relevant, although a randomized controlled trial would have been ideal. However, at the current state of knowledge in the care of gastric cancer patients, a corresponding study design can be of value to generate a baseline platform for conducting a future randomized controlled trial. The psychological conditions of the patients, stage of cancer, type of cancer, site of the tumor, presence of metastasis, and comorbidities, etc., were not evaluated, which could influence the outcomes. For instance, T4 tumors may increase the difficulty of the operation and might induce a higher risk of complications. Patients undergoing neoadjuvant therapy comprised a small group. In nurse-led preoperative counseling cohort patients had surgery without a surgeon describing the operation. This is unusual.

## 5 Conclusion

The current study was a retrospective study on nurse-led vs surgeon-led vs no preoperative counseling and outcomes in gastric cancer surgery. A total of 405 patients who underwent radical gastrectomy for gastric cancer were included, and the main result was that nurse-led preoperative counseling was associated with fewer symptoms, pain, hospital stay, and mortality compared to other methods. Patients who undergo radical gastrectomy for gastric cancer require preoperative healthcare professional-led counseling and healthcare professional-led follow-up care. Preoperative healthcare professional-led care, especially nurse-led counseling and nurse-led postoperative follow-up care, could reduce the length of total hospital stays, immediate and late postoperative complications, and death events. However, the success of the treatment is “teamwork (surgeon, nurses, social workers, nutritionists, etc.).” Preoperative counseling by a healthcare professional is necessary in patients who have undergone radical gastrectomy for gastric cancer. The experience and expertise of surgeons and surgical techniques could lead to a decrease in late postoperative complications and death of patients after radical gastrectomy for gastric cancer. The absence of neoadjuvant chemotherapy was an independent parameter for worse postoperative complications and death. However, further evidence from prospective or randomized studies is required. The study provided a comparison of different methods of pre and postoperative counseling after radical gastrectomy, which is very important in providing care for patients with gastric cancer.

## Abbreviations

NC cohort: Patients received preoperative nurse-led counseling, postoperative nurse-led follow-up care between chemoradiotherapies and follow-up after radical gastrectomy, and received surgeon(s)’ visit every 3 months in follow-up, SC cohort: Patients received preoperative surgeon-led counseling before radical gastrectomy and surgeons visited every 3 months between chemoradiotherapies and during follow-up after radical gastrectomy, RC cohort: Patients did not receive preoperative professional-led counseling before radical gastrectomy and received surgeons visit every 3 months between chemoradiotherapies and during follow-up after radical gastrectomy, CI: confidence interval, PICU: postoperative intensive care unit, SD: standard deviation, Q1: 25th quartile value, Q3: 75th quartile value, ANOVA: Analysis of variance, *χ* ^2^-test: chi-squared test, MICU: medical intensive care unit.
